# Transcriptional Responses of the Bacterium *Burkholderia terrae* BS001 to the Fungal Host *Lyophyllum* sp. Strain Karsten under Soil-Mimicking Conditions

**DOI:** 10.1007/s00248-016-0885-7

**Published:** 2016-11-14

**Authors:** Irshad Ul Haq, Francisco Dini-Andreote, Jan Dirk van Elsas

**Affiliations:** Microbial Ecology Group, Groningen Institute of Evolutionary Life Sciences (GELIFES), University of Groningen, Nijenborgh 7, 9747 AG Groningen, The Netherlands

**Keywords:** Chemotaxis, Short-chain dehydrogenases, Oxidative stress, Metabolic potential, Bacterial-fungal interactions

## Abstract

In this study, the mycosphere isolate *Burkholderia terrae* BS001 was confronted with the soil fungus *Lyophyllum* sp. strain Karsten on soil extract agar plates in order to examine its transcriptional responses over time. At the initial stages of the experiment (T1—day 3; T2—day 5), contact between both partner organisms was absent, whereas in the final stage (T3—day 8), the two populations made intimate physical contact. Overall, a strong modulation of the strain BS001 gene expression patterns was found. First, the stationary-phase sigma factor RpoS, and numerous genes under its control, were strongly expressed as a response to the soil extract agar, and this extended over the whole temporal regime. In the system, *B. terrae* BS001 apparently perceived the presence of the fungal hyphae already at the early experimental stages (T1, T2), by strongly upregulating a suite of chemotaxis and flagellar motility genes. With respect to specific metabolism and energy generation, a picture of differential involvement in different metabolic routes was obtained. Initial (T1, T2) up- or downregulation of ethanolamine and mandelate uptake and utilization pathways was substituted by a strong investment, in the presence of the fungus, in the expression of putative metabolic gene clusters (T3). Specifically at T3, five clustered genes that are potentially involved in energy generation coupled to an oxidative stress response, and two genes encoding short-chain dehydrogenases/oxidoreductases (SDR), were highly upregulated. In contrast, the *dnaE2* gene (related to general stress response; encoding error-prone DNA polymerase) was transcriptionally downregulated at this stage. This study revealed that *B. terrae* BS001, from a stress-induced state, resulting from the soil extract agar milieu, responds positively to fungal hyphae that encroach upon it, in a temporally dynamic manner. The response is characterized by phases in which the modulation of (1) chemotaxis, (2) metabolic activity, and (3) oxidative stress responses are key mechanisms.

**Electronic supplementary material:**

The online version of this article (doi:10.1007/s00248-016-0885-7) contains supplementary material, which is available to authorized users.

## Introduction

The soil bacterium *Burkholderia terrae* BS001 was originally isolated on the basis of its capacity to interact with the basidiomycetous soil fungus *Lyophyllum* sp. strain Karsten [1]. There is mounting evidence for the contention that this interacting pair forms an ecologically relevant mutualism, which we previously have coined the *B. terrae* BS001-*Lyophyllum* sp. strain Karsten interactome [2]. A plethora of functions is presumed to be important in the processes that underlie the interactions between the two organisms [3]. Briefly, *B. terrae* BS001 was found to successfully migrate through the soil matrix along with the growing hyphae of *Lyophyllum* sp. strain Karsten [4]. Moreover, this bacterium has the capacity to induce the release of glycerol by the fungus and efficiently utilize it as a carbon and energy source [5]. Finally, strain BS001—upon confrontation with *Lyophyllum* sp. strain Karsten and several other fungi—established agglomerates of cells, i.e., “primitive” biofilms, around the mycelia of these fungi [1].

To shed light on the interaction between *B. terrae* BS001 and *Lyophyllum* sp. strain Karsten, the recently sequenced 11.5-Mb strain BS001 genome [3, 6] was investigated with respect to the presence of genetic systems that are potentially involved in the interaction. Indeed, a suite of potential “interactome” genetic systems was present in the *B. terrae* BS001 genome, whereas other systems were suggested to be relevant for the “free-living” modus [3]. On the basis of these findings, we hypothesized that strain BS001 might exhibit a lifestyle in soil that involves two phases: one characterized by survival as a “loner” and a second one in which perceiving the presence of (fungal) hosts, and responding to these, constitutes the key ecological strategy. Nazir et al. [1] recently indicated that *B. terrae* BS001 is a “generalist” mycosphere colonizer rather than a specialist organism, as it could associate with a suite of different soil fungi. Given this facet of the lifestyle of strain BS001, particular genetic systems may have arisen that allow it to efficiently interact with diverse fungal types [1]. Interestingly, Pion et al. [7] recently found that the fungus *Morchella crassipes* apparently “farms” a *Pseudomonas putida* strain, allowing it to disperse and concomitantly use fungal-released compounds. The bacterium, in return, increased the resistance to stress of the fungal mycelium [7]. Thus, soil-exploring saprotrophic fungi might indeed constitute hot spots for the activity and growth of bacteria that are endowed with systems that allow to explore the fungal-created novel niches [4]. However, such fungi may also bring about conditions of stress (in particular oxidative stress), like suggested for the fungi *Alternaria alternata* and *Fusarium solani* in their interactions with the soil bacterium *Burkholderia glathei* [8]. On another notice, *Collimonas fungivorans* Ter331, upon confrontation with *Aspergillus niger* N400, did not only utilize compounds provided by the fungus but also expressed genes responsible for the production of antifungal agents [9]. Finally, the ectomycorrhizal fungus *Laccaria bicolor* S238N was found to respond in different and quite complex ways to antagonistic, neutral and beneficial soil bacteria [10]. Notwithstanding this growing body of knowledge on bacterial-fungal interactions in soil settings, none of the aforementioned studies assessed the behavior of bacteria with well-characterized genomes that contain—next to a core genome—a very large accessory part (such as *B. terrae* BS001) [3].

Here, we examined the transcriptional responses of *B. terrae* BS001 to the fungus *Lyophyllum* sp. strain Karsten. We hypothesized that time-dependent and dynamic responses might occur of the genetic systems that are of immediate interest to the ecological fitness of the bacterial partner of soil-exploring fungi upon confrontation with the fungus. Thus, to understand the responses that take place in the (largely carbon-limited) soil, we interrogated the transcriptional “null” status of the bacterium under such conditions. We then specifically investigated the transcriptional response of strain BS001 cell populations to the developing mycelium of *Lyophyllum* sp. strain Karsten in dual-culture systems on soil extract agar plates, using as controls fungus-less systems.

## Materials and Methods

### Strains, Culture Conditions, and Bacterial-Fungal Interactome

*Burkholderia terrae* BS001 maintained in the −80 °C culture collection was grown overnight in Luria-Bertani (LB) medium in a shaking incubator at 28 °C. The overnight culture was centrifuged, and the bacterial pellet washed twice with 0.85 % NaCl solution. Cultures yielding an estimated 4 × 10^8^ CFU mL^−1^ were thus used for the bacterial-fungal confrontation assay on soil extract agar (see below). For the B treatment, aliquots of 25 μL were streaked in five handlings in a straight 4 mm wide line using a sterile inoculation loop onto soil extract agar plates, establishing populations of around 1 × 10^7^ cells. For the B + F treatment, four plugs of *Lyophyllum* sp. strain Karsten grown on oat flake agar (OFA) medium were placed next to the bacterial streak line in parallel, separating both by a distance of 15 mm. For both treatments B and B + F, triplicates were used.

### Soil Extract Agar

We used soil extract agar (SEA) to establish the in vitro interactome. To prepare the soil extract, we used a loamy sand soil (organic matter 5 % and pH 5) [11] sampled in a field in Buinen, The Netherlands. For making the extract, 500 g of soil was taken up in 1 L of sterilized MilliQ water and vigorously shaken for 24 h (room temperature). Soil particles were removed by centrifugation at maximum speed (5430R Eppendorf centrifuge, Hamburg, Germany), after which the supernatant was filtered using folded qualitative filter paper (VWR European) and stored at 4 °C. To prepare 1 L of medium, 500 mL of soil extract, 0.5 g of yeast extract and 15 g of agar were mixed with 500 mL of MilliQ water. The pH of the medium was adjusted to 6.8 and the medium autoclaved. The SEA plates were prepared with ca. 22 mL of molten medium per plate.

### Soil Extract Analyses

Soil extract analyses were performed at NIOZ, Yerseke, The Netherlands, using a set of standard techniques (Nutrient Analyzers - Skalar and Seal, Southampton, UK). The soil extract contained 0.46 mM N-NH_4_, 0.00575 mM N-NO_2_, 1.67 mM N-NO_3_ and 1.67 mM N-NOx. The amounts of P-PO_4_ and Si-SiO_2_ in the extract were 0.09 and 0.15 mM, respectively. The extract also contained ca. 0.01 % of soil-extracted total organic carbon.

### Bacterial RNA Extraction and Sequencing

At each sampling time, the entire bacterial biomass was retrieved (using a sterilized spatula) from the SEA plates and resuspended in TRIzol-chloroform (900:150 μL), after which the mixture was bead-beaten for 40 s. Total RNA was then extracted using the TRIzol Reagent (Life Technologies, Carlsbad, CA, USA) according to the manufacturer’s instruction. Residual DNA was digested and removed using the TURBO DNA-free Kit (Life Technologies, Carlsbad, CA, USA) according to the instructions of the manufacturer. To remove ribosomal RNA, the MICROB*Express* Kit (Life Technologies, Carlsbad, CA, USA) was used according to the manufacturer’s instructions. Complementary DNA (cDNA)-based library preparation and sequencing using the Illumina MiSeq (250 bp paired-end sequencing run) platform were performed at Macrogen Inc., Seoul, South Korea.

### Transcriptome Sequence Analysis

We employed the bioinformatics pipeline implemented in the MicroScope interface [12], to analyze the raw sequence data and to perform the mapping and statistical analyses. The pipeline is a “Master” shell script composed of different parts (collection of Shell/Perl/R scripts). The quality of the sequence data was assessed by quality checks and read trimming was applied. The reads were then mapped onto the genome of *B. terrae* BS001 (hosted at MicroScope: http://www.genoscope.cns.fr/agc/microscope/home/) using the “SSAHA2” package [13]. This package identifies regions of high similarity using the SSAHA searching algorithm and aligns these by implementing cross-match sequence alignment [14] based on the banded Smith-Waterman-Gotoh algorithm [15]. For a hit to be retained, an alignment score equal to half of the read (at least) was required. The risk of false positives was reduced by extracting reliable alignments from SAM-formatted files using SAMtools (v.0.1.8) [16]. The “Bioconductor-Genomic features” package [17] was implemented to calculate the number of reads matching coding sequences [genomic objects] of the *B. terrae* BS001 genome. Differential gene expression between treatments was assessed using the Bioconductor-DESeq package [18] with default parameters. The DESeq normalized values of genes across replicates were *Z*-score standardized and visualized as heatmaps. All sequences are available in the short read archive (SRA) of the National Center for Biotechnology Information (NCBI), under the accession number SRP056279 and project number PRJNA278110.

As a criterion for our conclusions, with respect to differences in gene expression levels (reflected in the major conclusions of this study), we used the criterion of having ≥10 read counts, on average, per messenger type, with presence in all replicates. However, lower read counts were also considered in cases where the temporal development of gene expression was followed. We flagged these in the figures and placed a note of caution in the text, where needed. Care must thus be taken in the interpretation of the section concerning the detailed view of differentially expressed genes at T1, T2, and T3, because of incidental low read counts.

### Reverse Transcription Quantitative Polymerase Chain Reaction (RT-qPCR)

#### First Strand cDNA Synthesis

For cDNA synthesis, the SuperScript III first-strand synthesis system was used. Briefly, in a 0.5-μL tube, 150 ng of total RNA was placed (1.2 μL) and 1 μL random hexamers and dNTPs (10 mM) each were added to it. The volume was brought to 10 μL by adding RNase free water. It was incubated for 5 min at 65 °C and was subsequently placed on ice for at least 1 min. A cDNA synthesis mix was prepared by combining 2 μL 10× RT buffer, 4 μL 25 mM MgCl_2_, 2 μL 0.1 mM DTT, 1 μL RNase OUT (40 U/μL), and 1 μL SuperSript III RT (200 U/μL). To each RNA/primer mixture, 10 μL of cDNA synthesis mix was added and the final mixture was incubated for 10 min at 25 °C followed by 50 min at 50 °C. F inally, the reaction was terminated by incubation at 85 °C for 5 min. One microliter of RNase H was added to each reaction and incubated at 37 °C for 20 min. The cDNA was stored at −20 °C.

#### Primer Design and Quantitative Real-Time PCR

Using Clone Manager Suite (Sci-Ed software, Durham, NC, USA) with default parameters, gene-specific primers were designed to amplify regions of genes that had come up in the transcriptome analyses as being key to several aspects of the bacterial behavior in the system (selected genes: AKAUv1_790006 [*cheA*; chemotaxis], AKAUv1_2490031 [SDR; metabolism], AKAUv1_2490033 [NAD-dependent sugar epimerase/dehydratase; metabolism], and AKAUv1_2870060 (conserved exported protein, proxy of five-gene cluster; putative energy generation), ranging from 127 to 199 bp. The specificity of the primers for their respective targets was first tested using conventional gradient PCR and subsequent gel electrophoresis of the amplicons. The efficiency of the primers was then tested by real-time qPCR using purified PCR fragments as templates. Using 10-fold dilution series, standard curves were generated for each gene. The efficiency of each primer and the coefficient of determination (*r* ^2^) were calculated from the slopes of their respective standard curves. All quantitative RT-qPCR reactions were performed on an ABI Prism 7300 Cycler (Applied Biosystems, Frankfurt, Germany) using Power SYBRR Green PCR Master Mix (Applied Biosystems). For each biological replicate, 10 μL of Power SYBR Green Master mix, 1 μL each of forward and reverse primer, and 2 μL of 1:3 diluted cDNA in a final reaction volume of 20 μL were used. The amplification was carried out in the following steps: 50 °C for 2 min, 95 °C for 10 min, 40 cycles of 95 °C for 15 s, 60 °C for 20 s, and 72 °C for 30 s. Each reaction was carried out in triplicate for each of the three biological replicates of each sample at each time point (day 3, day 5, and day 8). Based on our RNA-sequencing data, the *rpoA* gene was chosen for normalization of the quantitative RT-PCR data. Relative expression values of each gene were determined using the comparative CT method 2^−ΔΔCT^ [19].

*T* tests were carried out using RStudio Version 0.99.893 – © 2009-2016 RStudio, Inc.

## Results

### Biomass Development of *Burkholderia terrae* BS)001 and *Lyophyllum* sp. strain Karsten on Soil Extract Agar (SEA) Plates

As from its introduction onto the SEA plates, *B. terrae* strain BS001 progressively developed biomass over the time of the experiment in both systems (B—bacterial strain alone and B + F—bacterial strain plus fungal inoculum). In the B + F system, fungal biomass slowly and progressively encroached upon the bacterial stripes, establishing strong physical contact at day 8. At three time points (T1—day 3, T2—day 5, and T3—day 8), the total bacterial biomasses from the B and B + F systems were sampled and subjected to bacterial RNA extraction, cDNA synthesis and high-throughput sequencing. This yielded a one-sided analysis of the transcriptional responses of *B. terrae* BS001–when under a (soil-relevant) “null” condition—to the presence of the fungal partner organism under two conditions: physically separated (T1, T2) or in contact (T3).

### Establishment of the *B. terrae* BS001 Transcriptome

Overall, 31,831,926 cDNA sequences were produced across all replicate samples (Table S1). Following sequence quality trimming and selection of the strain BS001 transcripts, a total of 5,972,111 cleaned reads was obtained (representing predicted CDSs only). This was taken as the initial dataset that was used for all downstream analyses. The raw read counts of the genes across all treatments (B and B + F) at all time points (day 3, day 5 and day 8) are provided in Table S2. At the level of COG (clusters of orthologous genes) classes, representatives of all broad functional categories were found at T1, T2 and T3, in both treatments. The distribution of reads across the COG classes is provided in Fig. S1A and S1B. Moreover, a global visualization of the differentially expressed genes of *B. terrae* BS001 upon confrontation with *Lyophyllum* sp. strain Karsten is shown in Fig. S2.

The collective data revealed a very dynamic global transcriptional response of strain BS001 as a response to the SEA medium, over the whole temporal regime, and this dynamism was also found in the systems with *Lyophyllum* sp. strain Karsten. The transcriptional landscape was typified by: (1) a generic response to the SEA conditions and (2) limited sets of genes being responsive to the fungus. Both types of responses were different between the physical-contact (T3) versus no-contact (T1 and T2) stages, which is explored in greater detail in the following.

### Analysis of the Transcriptome of Strain BS001 Reveals a (Generic) Stress Response on SEA and Modulation of the Response by the Presence of *Lyophyllum* sp. Strain Karsten

**Fig. 1:**
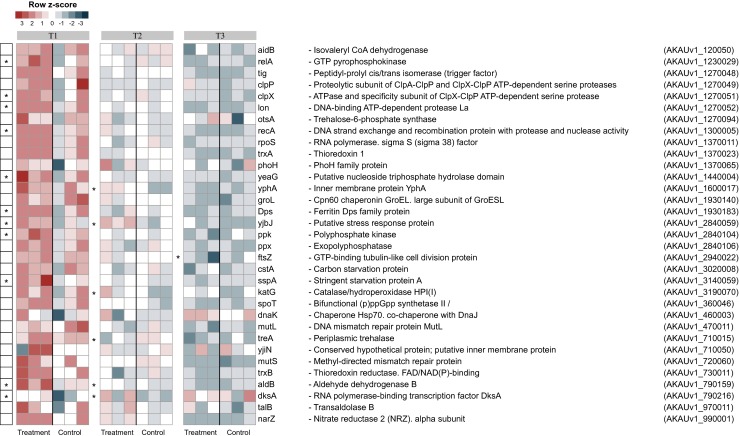
Heat map showing the expression level of selected stress response-related genes. The selected stress-related genes refer to the B (*B. terrae* BS001) and B + F (*B. terrae* BS001 + *Lyophyllum* sp. strain Karsten) treatments at time points *T1* (day 3), *T2* (day 5), and *T3* (day 8). *Statistically significant changes (statistical analysis was performed using DESeq; *P* < 0.05) between B and B + F treatments. The heat map was constructed based on normalized read counts. The standardized normalized read count, denoted as the row *Z*-score, is plotted in color scale (*red* indicates higher expression and *blue* indicates lower expression). The putative gene products are given in front of each gene with their respective locus tags

A first key observation was that the cells of strain BS001 were, apparently, in a state of (starvation) stress on the SEA medium, from T1 through T3 (Fig. 1). Hence, the predicted (alternative sigma factor) RpoS-encoding gene AKAUv1_1370011 was dynamically expressed in both treatments, with a trend (albeit not significant, *P* > 0.05) of raised expression at the fungus (Fig. 1). Moreover, homologs of (as per the *Escherichia coli* annotation) the RpoS-regulated genes [20] *katG* and *otsA* (cellular “processes”) were expressed similarly across all three time points (Fig. 1). Other transcripts for stress response-relevant proteins produced in both treatments were those of *groEL*, *dnaK*, *recA*, *ftsZ*, *mutL* and *mutS* homologs. They were likely expressed in all conditions because they represent a set of housekeeping genes. Similarly, transcripts of homologs of *narZ* (nitrate reductase Z; energy metabolism), *aldB* and *treA* (carbon compound metabolism), *talA* (central intermediary metabolism), and *aidB* (DNA replication/repair) and homologs of *yeaG*, *yjbJ*, *yjiN* and *yphA* (producing as-yet-uncharacterized proteins) were detected in both treatments. Moreover, a gene *phoH* homolog, which was predicted to encode the phosphate starvation-inducible PhoH protein, had a similar expression pattern (Fig. 1).

Interestingly, gene *sspA*, which was predicted to encode the “stringent starvation protein” SspA, revealed a differential upregulation at T1 (log2 fold change 1.01; *P <* 0.05) (Fig. 1), whereas at later stages (T2: B, 29, B + F, 28; T3: B: 21, B + F: 18), its expression remained similar across treatments. Supporting the notion that the cells were under carbon starvation stress, gene *cstA* (AKAUv1_3020008; encoding “carbon starvation protein A”) also revealed initially high expression, which gradually subsided (Fig. 1).

### Partial Alleviation of Stress at Later Stages of the Interaction

**Fig. 2:**
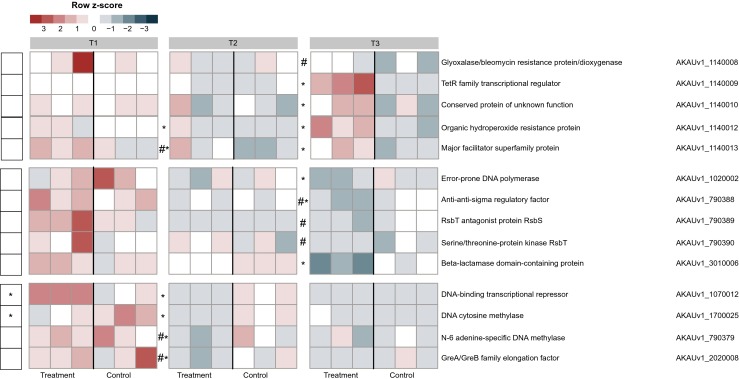
Heat map showing the expression levels of genes possibly related to stress alleviation. The data refer to genes in the B (*B. terrae* BS001) and B + F (*B. terrae* BS001 + *Lyophyllum* sp. strain Karsten) treatments at time points *T1* (day 3), *T2* (day 5), and *T3* (day 8). The heat map was constructed based on normalized read counts. The standardized normalized read count, denoted as the row *Z*-score, is plotted in color scale (higher expression is indicated by *red* color and lower expression by *blue*). The putative gene products are given in front of each gene with their respective locus tags. *Statistical significance (statistical analysis was performed using DESeq; *P* < 0.05) between the control and treatment. ^#^Genes with low read counts (see the “Materials and Methods” section)

A small set of genes was found to be expressed to somewhat similar levels at T1, with subsequent different expression levels in B + F at T2 and T3. Some of these genes were upregulated as a result of a potential oxidative stress response while others were downregulated (albeit based on low read counts for some of the genes; Fig. 2). These genes, which were all predicted to be involved in cellular responses under stress, included homologs of *rsbR* and *rsbS*, which encode “activator of sigma-B (RsbR) and anti-RsbT (RsbS),” respectively. The RsbR and RsbS proteins may play roles in the response of *B. terrae* BS001 to nutritional/environmental stresses such as salt, heat, acid or ethanol [21]. Surprisingly, a gene, AKAUv1_1020002, encoding error-prone DNA polymerase *dnaE2* (log2 fold change −2.03; Fig. 2), was expressed throughout and then downregulated at the physical-contact stage with the fungus. Given that error-prone polymerases enhance mutagenesis under starvation stress [22, 23], this might suggest partial alleviation of starvation (or other) stress. Similarly, gene AKAUv1_3010006 (encoding a metallo-beta lactamase domain protein; function unknown) was also downregulated (log2 fold change −1.73). Interestingly, at T2, another gene, AKAUv1_1070012 (encoding a homolog of a DNA-binding transcription antiterminator with a cold shock domain), was downregulated (log2 fold change −1.63) (Fig. 2). The gene product may regulate chromosome condensation and antitermination of transcription [24, 25], cold shock response [26], and even modulate RpoS [27]. Notably, gene AKAUv1_1700025 (encoding a DNA cytosine methylase) was also downregulated (log2 fold change −1.68) at T2. In *E. coli*, such a gene modulates (limits) the expression of ribosomal protein genes during stationary phase [28] and the expression of the *rpoS* gene [29]. Genes AKAUv1_790379 (encoding adenine specific DNA methylase; log2 fold change −2.09; low read counts) and AKAUv1_2020008 (encoding GreA/GreB elongation factor; log2 fold change −2.40) were also downregulated (low read counts; Fig. 2). The latter protein is known to have chaperone activity and resolve the undesirable aggregation of proteins [30].

However, the *ohr* gene (AKAUv1_1140012; log2 fold change 2.05), which encodes an “organic hydroperoxide resistance” protein—a key organic peroxide scavenger [31]—was upregulated (Fig. 2). In *Shewanella oneidensis*, a similar protein scavenges organic peroxides (tertiary butyl hydroperoxide) [32], thus being a key element in the oxidative stress response.

### Chemotaxis is a Prime Response of *B. terrae* BS001 on SEA to the Presence of Lyophyllum sp. Strain Karsten

**Fig. 3:**
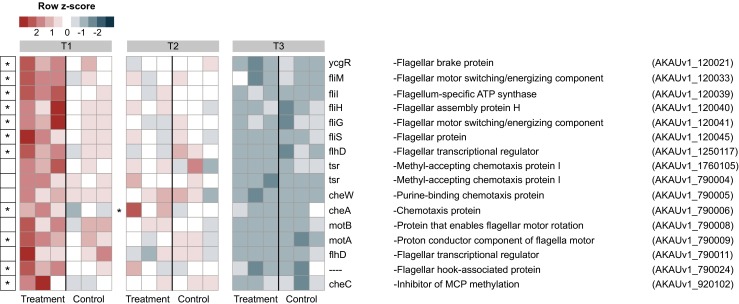
Heat map showing the expression patterns of chemotaxis and flagellar motility related genes. The data refer to genes in the control (*B. terrae* BS001) and treatment (*B. terrae* BS001 + *Lyophyllum* sp. strain Karsten) at time points *T1* (day 3), *T2* (day 5) and *T3* (day 8). *Statistical significance (statistical analysis was performed using DESeq: *P* < 0.05) between control and treatment. The heat map was constructed based on normalized read counts. The standardized normalized read count, denoted as the row *Z*-score, is plotted in color scale (*red* indicates higher expression and *blue* indicates lower expression). The putative products are given in front of each gene with their respective locus tags

At both “no-physical-contact” phases T1 and T2, the expression of a suite of genes, classed to COG class N (encompassing genes for cell motility and flagellar movement, next to secretion systems of types 2 and 3), was significantly enhanced in the B + F as compared to the B treatment. In particular, the expression of the *cheA* gene (AKAUv1_790006; histidine autokinase, assisting in the onset of chemotaxis) was significantly (*P <* 0.05) raised (Fig. 3). Similarly, fungal-incited upregulation of other chemotaxis-related genes, i.e., *cheW*, *cheC*, *motA*, *motB*, and “chemotaxis-related” (*tsr*) genes AKAUv1_1760105 and AKAUv1_790004 (both encoding “methyl-accepting chemotaxis protein I”) was observed, most strongly at the no-physical-contact stages (Fig. 3; significance at *P <* 0.05 indicated by *). Concurrent with this upregulation, homologs of the flagellar biosynthesis genes *flhD*, *fliS*, *fliM*, *fliG*, *fliH*, *flil* and *ycgR* were also significantly upregulated (*P <* 0.05).

In the light of the potential importance of protein secretion systems [2, 3] in the bacterial-fungal interactome, we then examined the expression levels of type-2, type-3 and type-6 secretion systems (T2SS, T3SS, T6SS). Only low expression levels were found for the genes in these systems across both treatments. However, the genes in the whole T6SS cluster 1 [3] (log2 fold increase 0.40–0.74) were upregulated at T1, with the differences for ten genes being statistically significant (*P* < 0.05) (Table S3). Moreover, gene AKAUv1_2840083 (encoding a lytic transglycosylase) was also expressed in both treatments over the whole, with a slight upregulation in the B + F treatment at T1 (log2 fold change 0.51; Table S3).

### Metabolic Responses of *B. terrae* BS001 Occur Dynamically and Differentially at *Lyophyllum* sp. Strain Karsten

**Fig. 4:**
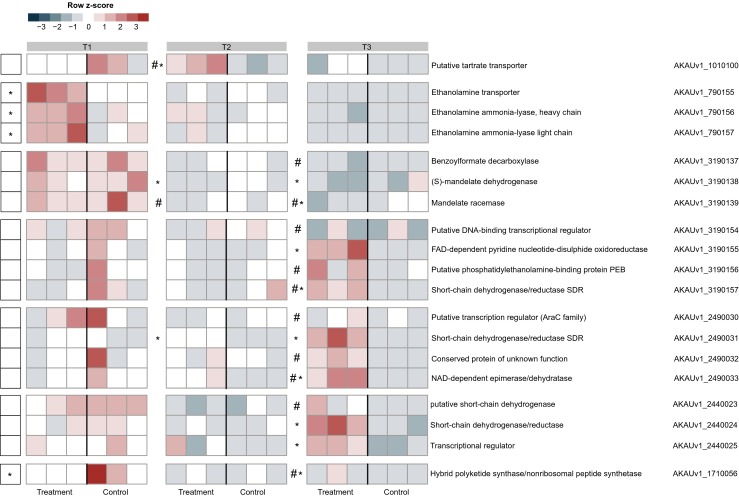
Heat map showing the expression patterns of gene clusters involved in diverse metabolic and energy generation pathways. The data refer to genes in the control (*B. terrae* BS001) and treatment (*B. terrae* BS001 + *Lyophyllum* sp. strain Karsten) at time points *T1* (day 3), *T2* (day 5), and *T3* (day 8). *Statistical significance (statistical analysis was performed using DESeq; *P* < 0.05) between control and treatment. The heat map was constructed based on normalized read counts. The standardized normalized read count, denoted as the row *Z*-score, is plotted in color scale (*red* indicates higher expression and *blue* indicates lower expression). The putative products are given in front of each gene with their respective locus tags. ^#^Genes with low read counts (see the “Materials and Methods” section)

With respect to putative metabolic up- and/or downshifts in *B. terrae* BS001, a dynamic picture of gene expression was obtained that pointed to a time-dependent metabolic response to (1) the SEA and (2) the presence of fungal hyphae (Fig. 4).

Probably as a response to a small metabolite secreted by *Lyophyllum* sp. strain Karsten into the SEA at T1, we observed a strong upregulation of three clustered genes (AKAUv1_790155, AKAUv1_790156 and AKAUv1_790157) that are predicted to encode an ethanolamine transporter and the large and small subunits of ethanolamine ammonia lyase, respectively, and are possibly involved in the transport and metabolism of compounds like ethanolamine (Fig. 4). However, this differential response evened out quite dramatically at T2 and T3. In contrast, three contiguous genes, AKAUv1_3190137, AKAUv1_3190138 and AKAUv1_3190139, predicted to encode proteins involved in the metabolism of the aromatic soil compound mandelate were highly expressed at T1 in the B, but less so in the B + F treatment (Fig. 4), whereas at T2 and T3, this response diminished significantly (low count data; Fig. 4). Interestingly, gene AKAUv1_1010100, which is predicted to encode a putative tartrate transporter, was downregulated at T1 and upregulated at T2 (low count data; Fig. 4), indicating the potential utilization of a tartrate-like compound initially from the SEA and later from the fungus.

Several other genes, collectively belonging to two different clusters and possibly involved in metabolic routes for energy generation, were upregulated at T2 and T3, but not at T1 (Fig. 4). Notably, these clusters each contained at least one gene that was predicted to encode a short-chain dehydrogenase (SDR), which suggested they were part of energy generation modules. SDRs are known to catalyze the oxidation (or reduction) of sugars, alcohols, steroids, diverse xenobiotics and aromatic compounds in an NADP(H)-dependent fashion [33]. One such gene, AKAUv1_2490031 (also validated by RT-qPCR; see later), was upregulated at T2 and more strongly so (log2 fold change 4.64) at T3 (Fig. 4). The downstream gene AKAUv1_2490033 (encoding an NAD-dependent sugar epimerase/dehydratase, probably involved in the conversion of UDP-α-d-glucose to UDP-α-d-galactose; log2 fold change 2.84; also validated by RT-qPCR) was also upregulated (low read counts; Fig. 4), whereas the intervening gene (AKAUv1_2490032) encoding a conserved hypothetical protein, as well as the up- and downstream genes did not show differential expression. In a second cluster, two SDR-encoding genes (AKAUv1_2440023; AKAUv1_2440024), next to one encoding a transcriptional regulator (AKAUv1_2440025), were also upregulated. Interestingly, gene AKAUv1_2440024 (log2 fold change 2.69) had a keto reductase domain. A third cluster encompassed genes AKAUv1_3190155, AKAUv1_3190156 and AKAUv1_3190157 (encoding a putative FAD-dependent pyridine nucleotide-disulfide oxidoreductase (log2 fold change 3.22), a putative phosphatidylethanolamine binding protein, and a short-chain dehydrogenase/reductase, respectively, was found to be expressed throughout, albeit at low read counts (Fig. 4).

### A Five-Gene Cluster with Relevance for Energy Generation Unveiled

**Fig. 5:**
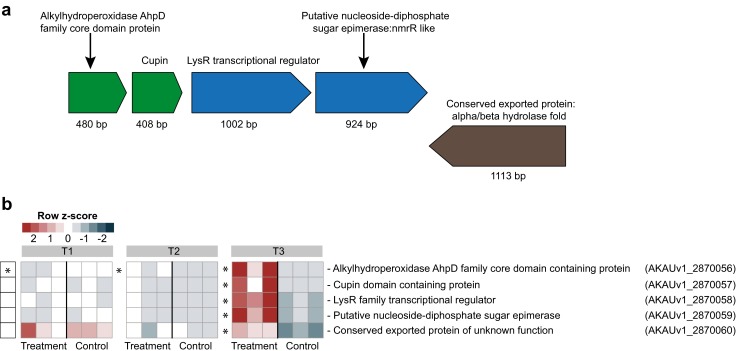
Heat map showing the genetic organization and expression pattern of a gene cluster of *B. terrae* BS001 involved in presumed metabolism and energy generation. **a** The genetic organization of the cluster of genes that significantly upregulated at T3 (day 8). **b** Heat map of the expression patterns of the genes in the control (*B. terrae* BS001) and treatment (*B. terrae* BS001 + *Lyophyllum* sp. strain Karsten) at time points T1 (day 3), T2 (day 5) and T3 (day 8). *Statistical significance (statistical analysis was performed using DESeq; *P* < 0.05) between control and treatment. The heat map was constructed based on normalized read counts. The standardized normalized read count, denoted as the row *Z*-score, is plotted in color scale (*red* indicates higher expression and *blue* indicates lower expression). The putative products are given in front of each gene with their respective locus tags

With respect to metabolism and energy generation, five clustered genes, AKAUv1_2870056 through AKAUv1_2870060, were dynamically modulated and significantly upregulated at T3. BLAST-N searches of the whole region revealed nucleotide identities of 95 % (coverage of 91 %) to a similar region of the *B. caribiensis* MBA4 2,555,069 bp replicon. The first four genes of the cluster are located on the same strand and represent an operon (Fig. 5a), as predicted using Rockhopper [34, 35]. Of this operon, the first gene encodes a predicted alkyl hydroperoxidase (AHP) (log2 fold change 4.51) (Fig. 5b) and the second one (log2 fold change 3.81) a protein that belongs to the cupin superfamily. These two gene products might reflect a combination of energy generation, e.g. from a small molecule such as oxalic acid, and a concomitant oxidative stress response, as has been found in the interaction of *Brachypodium distachyon* with *Fusarium graminearum* [36, 37]. The response is possibly regulated by a LysR family transcriptional regulator encoded by gene AKAUv1_2870058, which was also upregulated (log2fold change 4.10). The fourth upregulated gene, AKAUv1_2870059, was predicted to encode a putative nucleoside-diphosphate sugar epimerase (log2 fold change 4.28). Finally, the fifth gene of the cluster, AKAUv1_2870060, possibly encodes a conserved (exported) “alpha/beta hydrolase fold” protein. Using BLAST-P against the ESTHER database, we found that the predicted protein was ca. 60 % similar to a poly(aspartate) hydrolase from *B. glumae*.

### Oxalate Metabolism and Energy Generation

Several gene clusters with putative relevance for the uptake and utilization of oxalate were found to be dynamically modulated over time, in the B + F treatment. First, a cluster of genes containing an oxalate/formate antiporter (AKAUv1_1160010), oxalyl-CoA decarboxylase (AKAUv1_1160013) and two formyl-CoA transferases (AKAUv1_1160014; AKAUv1_1160019) was upregulated at T1 and T2 (Fig. S3). Moreover, two genes in another cluster, encoding an oxalate/formate antiporter (AKAUv1_2660032) and a formyl-CoA transferase (AKAUv1_2660028), were also upregulated at T1. Strain BS001 further showed the upregulation of genes of the glycerate pathway [38], encoding tartronic semialdehyde reductase (AKAUv1_2390002) and glyoxylate carboligase (AKAUv1_2390004), indicating further processing of oxalate.

### Glycerol Uptake and Utilization

Given its presumed importance as an accelerator of metabolism, we examined the expression of the glycerol uptake (GUP) gene AKAUv1_1930108 [3] across all treatments. Our analyses did not reveal any differential response, indicating the GUP trait had a minor impact, if any at all. Concerning the utilization of glycerol, we investigated the expression of gene AKAUv1_1300029 encoding glycerol kinase across treatments; however, we did not find any differential responses.

### RT-qPCR Validation of Expression of Selected Genes

**Fig. 6:**
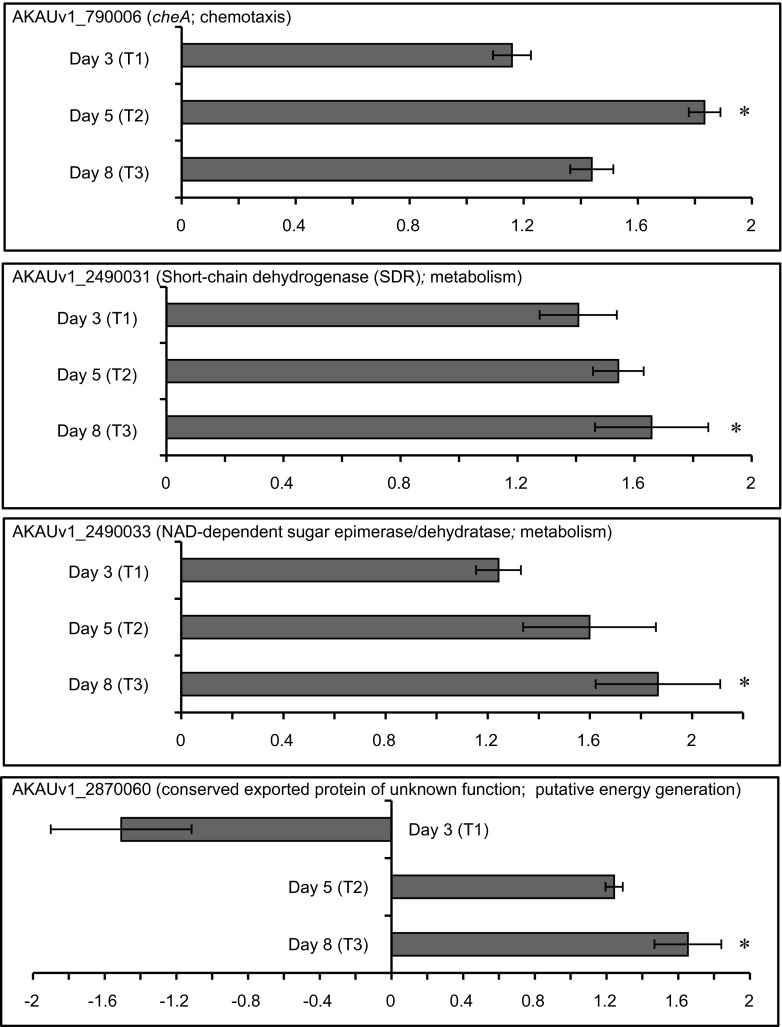
Bar charts representing the fold changes of selected genes at T1 (day 3), T2 (day 5) and T3 (day 8) in the treatment (B + F) relative to the control (B), obtained through RT-qPCR. *Statistical significance (*P <* 0.05; *t* test). The *error bars* represent the standard error of the mean

To validate our major conclusions drawn from the whole transcriptome analysis, relative expression analyses of four genes of *B. terrae* BS001 were performed using quantitative RT-qPCR at three time points (day 3, day 5 and day 8). The RT-qPCR generated results that validated the respective transcriptome data showed a similar trend at all time points (Fig. 6).

We observed that *cheA* was upregulated (fold change 1.83; *P <* 0.05) in the presence of *Lyophyllum* sp. strain Karsten at day 5. Similarly, at day 8, we noted upregulation (fold change 1.43; *P >* 0.05) of the *cheA* gene, in the B+F treatment (*B. terrae* BS001 and *Lyophyllum* sp. strain Karsten) compared to the B treatment, with *B. terrae* BS001 growing alone.

The expression of the short-chain dehydrogenase encoding gene (AKAUv1_2490031) had previously shown a pattern of upregulation at the physical-contact stage of the interaction. Our quantitative PCR data showed correlation with our initial observation, as we noted an upregulation (fold change: 1.54, *P >* 0.05 and 1.65, *P <* 0.05) in the expression of the gene at day 5 and day 8, respectively, in the presence of fungal mycelium.

The quantitative PCR data also revealed that the expression of NAD-dependent epimerase encoding gene AKAUv1_2490033 followed a similar trend as in the transcriptome analysis, with an upregulation at day 5 (fold change: 1.59, *P >* 0.05) and day 8 (1.86; *P <* 0.05) in the presence of fungal mycelium.

The gene AKAUv1_2870060 that presumably encodes a conserved hypothetical protein also showed a similar trend of upregulation at the physical-contact stage, as found in the transcriptome analyses. It was significantly upregulated by strain BS001 growing in the presence of the fungus, at the physical-contact stage (day 8; 1.65; *P <* 0.05). However, at day 5, it was only slightly upregulated (1.24; *P >* 0.05).

### Detailed View of Differentially Expressed Genes at T1, T2 and T3

We here provide a brief account of other differentially expressed genes at each time point, which were for the most part not discussed in the foregoing.

#### Differentially Expressed Genes at T1

A total of 651 genes was found to be differentially expressed between treatments B + F and B at T1 (Table S4), 584 of these differences were statistically significant (*P* < 0.05). Only 33 genes were upregulated, with the remainder (618) being downregulated.

#### Upregulated Genes

Thirteen of the 33 upregulated genes were assigned to COG classes R (3 genes), S (2), P (2) and M, N, T, L, E, and CHR (1 each), whereas the remaining (20) could not be assigned to any class (Table S4). As indicated in the foregoing, the chemotaxis regulatory gene (*cheA*; belonging to class “N”) was strongly upregulated. Furthermore, the COG class M gene AKAUv1_220060, which is predicted to encode a d-heptose-1-phosphate adenylyltransferase (DHPA), was upregulated (log2 fold change of 1.82; Table S4). DHPA is involved in the biosynthesis of the lipopolysaccharide (LPS) precursor ADP-heptose, potentially of the inner core LPS [39]. Then, gene AKAUv1_710037, which is predicted to encode a protein involved in the biosynthesis of pyrroloquinoline quinone (PQQ), was upregulated (log2 fold change 1.82; Table S4). PQQ is a cofactor involved in cellular processes such as phosphate solubilization and the scavenging of reactive oxygen species, as well as in stress responses, in *Pseudomonas* [40, 41]. Furthermore, genes for iron acquisition and storage, notably AKAUv1_2280031, AKAUv1_2280033, AKAUv1_2440020 and AKAUv1_2280030 (encoding respectively bacterioferritins and a TonB family protein), were upregulated (log2 fold changes 0.52, 0.64 and 0.74; *P* < 0.05; Table S3). Bacterioferritins sequester iron that may be toxic to cells and release it when iron becomes limiting [42]. Finally, gene AKAUv1_690018 and three other genes, which were predicted to encode peptidases involved in the maturation and processing of a peptide antibiotic (like microcin B17 in *E. coli* [43]) were upregulated (log2 fold change 0.89; Table S3).

#### Downregulated Genes

Table S4 lists the main downregulated genes. About 20 % of these (that is, 125 of 618) were predicted to encode conserved proteins of unknown function, whereas about 40 % (260 genes) represented various “core metabolism” enzymes. In addition, about 78 genes encoded various membrane-bound transporters and another 36 transcriptional regulators. With respect to the transporters, ATP-binding cassette (ABC) (23) and major facilitator superfamily (MFS) (11) class transporters were downregulated (Table S5). Notably, the *cusA* gene (log2 fold change −3.51), which encodes a metal (copper/silver) membrane efflux system, was strongly downregulated (Table S5). CusA is a member of the resistance-nodulation-cell division (RND) proton-driven cation antiporter/symporter family that is involved in the efflux of heavy metals [44]. Another 70 downregulated genes encoded proteins that make part of various KEGG metabolic pathways (Table S6). These pathways encompass the transformation of carbohydrates, amino acids and fatty acids. Here, glycolysis/gluconeogenesis, citrate cycling and pyruvate and amino acid metabolic processes stood out. In addition, several genes (Table S6) involved in methane, nitrogen and aromatic hydrocarbon transformation pathways were also downregulated.

Interestingly, downregulation (log2 fold change −1.61) of a hybrid polyketide synthase/nonribosomal peptide synthetase (PKS/NRPS; AKAUv1_1710056) gene [3] was found, suggesting that modulation of this specific bacterial function might be key to interaction with the fungal host (Fig. 4).

#### Differentially Expressed Genes at T2

A total of 155 genes was found to be differentially expressed at T2 (threshold, >1.5 log2 fold change). Of these, 66 were statistically significant (*P* < 0.05). Fifteen of the latter genes were upregulated and 51 downregulated in the B + F as compared to the B treatment (Fig. S4). These are described hereunder.

#### Upregulated Genes

As discussed above, the chemotaxis regulator *cheA* continued to be upregulated at T2, indicating a persisting response to stimuli from the fungus. However, the expression of other genes related to flagellar motility was not differential (Fig. 3). Interestingly, gene AKAUv1_1000027, which was predicted to encode a “Suppressor of variegation-Enhancer of zeste-Trithorax (SET)-domain containing protein” (log2 fold change 1.68; Fig. S4) was upregulated. This may suggest that the predicted 16.46-kDa SET domain protein, a potential T3SS-secreted effector, might be “early” induced, to serve as a potential modulator of fungal gene expression [45]. Notably, transcription of a gene for a putative oxidoreductase, AKAUv1_530022, was upregulated (log2 fold change 1.93), next to that of a gene for a putative cytochrome c552 (log2 fold change 2.83; AKAUv1_2920083) (Fig. S4). Another upregulated gene, AKAUv1_540146, encoding a predicted alcohol dehydrogenase (log2 fold change 1.91), indicated (diverse) metabolic processes were active. Moreover, a gene encoding lactoylglutathione lyase (glyoxalase I, possibly detoxifying the metabolic by-product methylglyoxal), AKAUv1_540002, next to gene AKAUv1_540003 (log2 fold change 1.35; *P* < 0.05), was also upregulated.

With respect to potential metabolism, two genes predicted to be involved in purine and valine/leucine metabolic pathways were upregulated (≥1.5 log2 fold change; Table S7). Gene AKAUv1_1780022 is predicted to encode an allantoicase with a potential role in the transformation of purines or the nitrogen-rich derivative allantoin [46], releasing nitrogen for anabolism [47, 48]. Moreover, the upregulated gene AKAUv1_3070011, that was predicted to encode 3-hydroxy isobutyrate dehydrogenase, might be involved in the valine/leucine degradation pathway (Table S7).

#### Downregulated Genes

Eight of the 51 downregulated genes at T2 were involved in central metabolic processes, with four being part of one KEGG pathway each (for glutathione, vitamin B6, glycerophospholipids and cysteine/methionine, respectively). The remaining four genes were potentially involved in more than one pathway, i.e., glycolysis/gluconeogenesis, glyoxylate/dicarboxylate, galactose and pentose/pyruvate (Table S7).

Interestingly, gene AKAUv1_2870089, predicted to encode a glutathione-dependent formaldehyde-activating protein (involved in the detoxification of formaldehyde [49]), was downregulated (log2 fold change −2.72), with its upstream gene AKAUv1_2870088, predicted to encode a methyltransferase, also being downregulated (log2 fold change −1.83; Fig. S4). In *Saccharomyces cerevisiae*, such genes are reported to play roles in responses to stress [50]. Furthermore, two other (contiguous) genes, AKAUv1_990014 and AKAUv1_990015 (both predicted to encode conserved proteins of unknown function), were strongly downregulated (log2 fold changes −2.34 and −2.99; Fig. S4). Secondary structure prediction of the latter protein revealed it to resemble a p-amino benzoate N-oxygenase (AurF) from *Streptomyces thioluteus*, which has a role in the oxidation of aromatic hydrocarbons such as aminoarenes to nitroarenes [51, 52].

Interestingly, gene AKAUv1_110198 (encoding a “conserved protein of unknown function”) was downregulated (log2 fold change −1.59). The predicted gene product was homologous (34 % amino acid identity) to the *purD* gene product (phosphoribosylamine-glycine ligase), which is involved in purine metabolism and is upregulated in response to butanol and butyrate stress in *Clostridium acetobutylicum* [53]. Moreover, the predicted ATP-dependent carboligase-encoding gene AKAUv1_110221 was also downregulated (log2 fold change −1.66).

#### Differentially Expressed Genes at T3

Upon physical contact of strain BS001 with the fungal hyphae at T3, a total of 136 bacterial genes was differentially expressed. Ninety-six of these differences were statistically significant (*P* < 0.05). Out of these 96 genes, 62 were upregulated and 34 downregulated (Fig. S5).

#### Upregulated Genes

Next to the metabolic genes discussed in the foregoing, three genes representing three KEGG metabolic pathways were upregulated at T3 (Table S8). The predicted proteins were likely involved in the transformation of aminoacyl-tRNA and of arginine/proline. Moreover, gene AKAUv1_2480024, encoding an enzyme of the chlorocyclohexane/chlorobenzene and toluene degradation pathway, was also upregulated (log2 fold change 1.57). Regarding ionic homeostasis, gene AKAUv1_2180021, encoding chloride channel protein EriC, an H^+^/Cl^−^ antiporter, was upregulated (log2 fold change 1.58; Fig. S5). The EriC protein may be involved in (acid) stress tolerance, as found in *E. coli* [54].

Moreover, the aforementioned PKS/NRPS biosynthetic gene AKAUv1_1710056 was upregulated at T3, albeit at low read counts (log2 fold change 1.68), indicating potential modulation of *Lyophyllum* sp. strain Karsten physiology upon physical contact. Finally, two genes, AKAUv1_2420001 and AKAUv1_2490034 (predicted to encode transposases), were also upregulated (log2 fold change 1.62 and 2.39). A similarly enhanced expression of the mobility of genetic elements was reported for *C. fungivorans* Ter331 in its interaction with *A. niger* [9].

#### Downregulated Genes

With respect to general KEGG metabolic pathways, the expression of six genes was downregulated (Table S8). These included two genes each, involved in purine and aminobenzoate metabolism, and one each in galactose and tryptophan metabolism. The gene AKAUv1_110190 (encoding coenzyme PQQ synthesis protein C) was downregulated (log2 fold change −1.90; *P* < 0.05; Fig. S5). In *Pseudomonas*, the *pqqC* protein is involved in the response to stress [41], being that in the rhizosphere of pine, *P. putida* KT2440 preferentially activates it [55]. Thus, the downregulation of the strain BS001 *pqqC* may relate to the alleviation of stress. Similarly, gene AKAUv1_2820103, predicted to encode a NodT family efflux transporter, was downregulated (log2 fold change −1.47). Similarly, the aforementioned (Fig. S5) methyltransferase encoding gene, AKAUv1_2870088, was also downregulated (log2 fold change −1.70).

## Discussion

So far, only few studies have unraveled the complex nature of bacterial-fungal interactions at the transcriptional level [8–10, 56, 57]. Recently, *C. fungivorans* strain 331 was shown to invest substantial cellular resources into the capacity to utilize compounds provided by its host fungus *A. niger*, as well as in the production of antifungal agents [9]. In the current study, *B. terrae* BS001 was interrogated with respect to its response to the fungus *Lyophyllum* sp. strain Karsten on SEA plates mimicking soil conditions. Under the selected conditions, heterotrophs such as *B. terrae* BS001 are expected to express responses to the scarce resources, in particular sources of carbon and energy. We hypothesized that the presence of fungal hyphae would drive additional responses, resulting in the potential exploration of fungal-derived resources, most likely along an approximation to, followed by a physical association with, the fungus. The existence of a physical association of *B. terrae* BS001 with *Lyophyllum* sp. strain Karsten was recently demonstrated using fluorescence microscopy [1].

### Gene Expression Patterns of *B. terrae* BS001 on the SEA Medium

The observations at T1 and T2 (no physical contact between partners) versus the physical contact phase T3 enabled the examination of the bacterial responses to either “distant” or “proximate” fungal hyphae. Thus, effects of highly diffusible (or even volatile) fungal-released compounds or of changes in the (nutritional) status of the medium versus those of more physical types of interaction, were assessed. Overall, upon introduction, strain BS001 was clearly confronted with (starvation) stress conditions on the SEA plates, which was consistent with the reduced amount of total carbon in the medium. Given that the stress-relevant transcript densities in the B + F treatments at T1 were initially as high as, or higher than, those in the B treatment, little, if any, relief of the stress by the fungus occurred at this time. Key to the contention that the bacterial population was under (generic) stress were the high expression levels of *rpoS* and numerous RpoS-driven genes. We hypothesized that, following initial bacterial growth, heterogeneous cell populations emerged on the SEA plates, with different levels of growth (frontier cells) versus stress (backward cells). Such situations very likely occur in the soil, where spatial constraints foster the coexistence of both growing and growth-arrested (starvation-stressed) cell populations. Similar observations have been reported for other bacterial systems [58, 59].

The high expression of genes encoding lytic transglycosylases in both treatments over time was intriguing. Such enzymes are possibly involved in cell wall recycling/turnover, cell division and insertion of membrane-spanning structures (i.e., secretion systems and flagella) [60, 61]. They are expressed under elevated stress [62, 63], degrading peptidoglycan and remodeling the cell wall [64]. It appears that, due to this action, strain BS001 probably restructured its cellular make-up, when confronted with the conditions of SEA, as a result of the nutrient-limited conditions.

### Early Gene Expression by *B. terrae* BS001 as a response to *Lyophyllum* sp. strain Karsten

The disproportionate expression of the *cheA* gene in the presence of the fungus at T1 as well as T2, highlighted the likely role of chemotaxis and flagellar motility in the early stages of interaction of *B. terrae* BS001 with *Lyophyllum* sp. strain Karsten. That these data indeed suggested a chemotactic response occurred was supported by the data obtained via RT-qPCR analysis of the *cheA* gene (Fig. 6). This analysis was striking, as at T3 no significant difference in the expression levels of the respective genes was observed between the treatments. Although migration of bacterial cells on the agar plate surface did not visibly take place, *Lyophyllum* sp. strain Karsten most likely released compounds into the SEA that acted as chemoattractants for *B. terrae* BS001. Hence, chemotaxis is likely central to the behavior of *B. terrae* BS001 in soil when confronted with a (distant) attracting fungus. In *Sinorhizobium meliloti*, chemotaxis also drives the movement of cells towards plant-secreted chemoattractants [65], whereas a study with *Fusarium oxysporum* identified fusaric acid as an attractant for *Pseudomonas fluorescens* WCS365 [66].

Rather unexpectedly, we also observed an upshift in the expression of the T6SS at T1. Indeed, the T6SS has been reported to mediate the bacterial response to oxidative stress [67], regulating RpoS, modulating general stress response regulators [68], and playing a role in osmotolerance and pH homeostasis [69, 70]. Thus, a rather early response to SEA-induced stress conditions was apparent.

### Metabolic Response and Energy Generation—Several Putative Compounds Implicated

The overall metabolic responses of *B. terrae* BS001 in the B and B + F treatments may be characterized by a quick depletion of the carbonaceous compounds present in the SEA, followed by a progressively stronger response to the fungus as a potential provider of such resources. Thus, multiple sets of genes for a suite of generic energy generation pathways, as well as the specific mandelate utilization pathway, were suppressed by the fungus. In *Pseudomonas*, mandelate/mandelic acid (derived from the soil metabolite amygdalin) can serve as carbon and energy sources [71, 72]. Next to repressed gene sets, others were highly expressed, indicating inductive events. For instance, the dynamic expression pattern of the ethanolamine (*eut*) utilization operon over time may indicate that ethanolamine or similar compounds were being actively captured and metabolized, up to their depletion from the medium. The molecule, much like ethanolamine, may have constituted a metabolic cue in the system, in a temporally defined manner [73, 74]. The expression of a putative tartrate transporter encoding gene was notable, as several (ectomycorrhizal) fungi have been found to release tartrate and other low-molecular-weight organic compounds under nutrient-poor conditions [75]. In *Rhizobium leguminosarum* biovar *viciae*, a similar protein acts in the utilization of tartrate in the rhizosphere of pea and alfalfa [76].

### Glycerol

Nazir et al. [5] previously reported that glycerol is a main compound that is released by *Lyophyllum* sp. strain Karsten in liquid systems, constituting a resource for *B. terrae* BS001. However, the GUP system, previously hypothesized to serve in the capturing of extracellular glycerol, as well as a glycerol kinase gene, were not significantly upregulated at the fungus. Although glycerol may have become available, it may have been usurped by passive diffusion across the membrane. Alternatively, the switch-on of the *gup* gene may have only occurred in the frontier cells that were faced with the highest glycerol levels, so that it did not stand out as significant in the overall analyses.

### Oxalate and Its Putative Transformation

The enhanced expression of several systems that are potentially involved in oxalate capture and transformation indicates that strain BS001 may assimilate fungal-released compounds such as oxalate [77] as sources of carbon and energy. Strain BS001 may exhibit a biphasic response mechanism towards oxalate in combination with other molecules that are released by the fungus, where genes for degradation are upregulated at earlier or later stages. A similar trend was reported for *C. fungivorans* Ter331 in its interaction with *A. niger* [9]. We hypothesized that gene AKAUv1_2870057 (encoding a protein of the cupin superfamily) has a role as an oxidase, potentially of released oxalate, thus catalyzing its breakdown to carbon dioxide and hydrogen peroxide. The effects of the resulting hydrogen peroxide may have been neutralized by a peroxidase encoded by the adjacent gene (AKAUv1_2870056). Apart from this, the expression of genes involved in the degradation of oxalate, both at earlier and later stages of the interaction, likely indicates a complex scenario, in which strain BS001 may switch on/off the expression of genes, subject to the availability of oxalate.

### Partial Relief of Stress by *Lyophyllum* sp. strain Karsten at T3

At the “physical-contact” phase T3, the stress-related genes *dnaE2*, *rsbR*, *rsbS*, *rsbT* and *pqqC* (although the latter at low read counts) and a gene (AKAUv1_2870088) encoding a methyltransferase showed a progressive lowering of expression, suggesting a—possibly partial—relief of the stress by the fungus. However, we are careful in our conclusion due to the fact that some of the data were supported by low read counts (see Fig. 2). This partial relief of stress by the presence of *Lyophyllum* sp. strain Karsten may be due to the delivery of palatable compounds such as glycerol and oxalate. Many fungi are known to release such compounds [5, 78]. For instance, a recent study on the interaction of *B. glathei* with *A. alternata* and *F. solani* reported an attenuation of bacterial stress due to phosphate and carbon (SspA and CstA) starvation as a result of fungal presence [8]. We surmised that, in addition to the general SEA-incited (nutrient) stress, competition for nutrients in the interactome may have been fierce at T1, whereas the two partners differentiated the niche as the interaction progressed to physical contact.

### An Oxidative “Burst” or Bacterial Metabolism-Generated Oxidative Stress

Given that strain BS001 revealed fungal-incited upregulation of several genes involved in the oxidative stress response, in particular at T3, either the fungus or the bacterium itself may have produced H_2_O_2_ as a metabolic by-product. The latter is known to occur in the oxalate oxidative pathway. Possibly, a partial alleviation of medium-induced stress resulted in metabolism concomitant with an enhancement of oxidative stress. This effect has also been observed in the interaction of *B. glathei* with *A. alternata* and *F. solani* [8]. Indeed, metabolic activities were clearly enhanced at T3, and the SDRs expressed match the expectation of their involvement in energy production by strain BS001 from fungal-released substrates. RT-qPCR analysis of the genes encoding SDR (AKAUv1_2490031), NAD-dependent epimerase (AKAUv1_2490033), and conserved hypothetical protein (AKAUv1_2870060), validated the expression patterns revealed by the whole transcriptome analyses (Fig. 6). In *P. fluorescens* BBc6R8, SDR-like proteins were recently shown to be upregulated in the presence of *L. bicolor* S238N [10]. Moreover, SDR mutants of *S. meliloti* have deficiencies in the catabolism of particular carbonaceous compounds, affecting its symbiosis with *Medicago sativa* [79].

### Potential Role for a *B. terrae* BS001 Secondary Metabolite

The downregulation of the hybrid PKS/NRPS gene at T1—in contrast to T3—in the presence of the fungus was interesting. It is possible that perception of the fungal hyphae at a distance allowed subpopulations of the strain BS001 cells to divert energy into the repression of production of the antifungal compound, thus allowing the fungus to get physically close to the bacterial growth. Upon physical contact, upregulation occurred (albeit at low read counts), by which the bacterium possibly modulated the fungal mode of growth to its own benefit. However, the nature of the natural product synthesized by the gene cluster is as yet unknown. *B. rhizoxinica*, an endosymbiont of the fungus *Rhizopus microsporus*, produces the secondary metabolite “rhizoxin” that acts on rice seedling cells, destroying these [80]. A large PKS/NRPS operon was found to be involved in rhizoxin biosynthesis [81]. We suggest that *B. terrae* BS001 expresses the NRPS/PKS gene cluster, under nutrient-limited conditions, in a different manner according with the relative “sphere of influence” of its fungal associate.

## Conclusion

Overall, our analyses reveal that the interplay between *B. terrae* BS001 and *Lyophyllum* sp. strain Karsten under soil-mimicking conditions is highly complex and dynamic. Clearly, *B. terrae* BS001 encounters stress conditions on the SEA medium used early on in the experiment, with several genetic systems, including chemotaxis and flagellar motility, being responsive to the fungal hyphae, perceived at a distance. The early responses also included some metabolic up- and downshifts, which is probably in line with the resources encountered in the system without/with the fungus. Then, the organism likely entered a different physiological state upon contact with the fungus, in which limited sets of particular metabolic genes, next to oxidative stress responsive genes, were activated, at the expense of other metabolic genes. On the basis of this observational study, it is clear that a more focused insight into each of the mechanisms underlying the interaction of *B. terrae* BS001 with its host fungus is required. Possibly, mutational analyses of the key genetic systems unveiled here should be combined with specific transcriptome and metabolic profiling approaches.

## Electronic supplementary material

Below is the link to the electronic supplementary material.

Fig. S1 (PDF 397 kb)

Fig. S2 (PDF 228 kb)

Fig. S3 (PDF 266 kb)

Fig. S4 (PDF 377 kb)

Fig. S5 (PDF 360 kb)

Table S1 (XLS 37 kb)

Table S2 (XLSX 6816 kb)

Table S3 (XLS 32 kb)

Table S4 (XLS 145 kb)

Table S5 (XLS 41 kb)

Table S6 (XLS 44 kb)

Table S7 (XLS 28 kb)

Table S8 (XLS 27 kb)
