# Supplementary material for: Transcriptional Responses of the Bacterium Burkholderia terrae BS001 to the Fungal Host Lyophyllum sp. Strain Karsten under Soil-Mimicking Conditions
Source: Microb Ecol. 2016 Nov 14;73(1):236–52. doi: 10.1007/s00248-016-0885-7 (PMC5209427; doi:10.1007/s00248-016-0885-7)
Supplement: Supplementary file 1 — (PDF 397 kb) [file 248_2016_885_MOESM1_ESM.pdf]

Transcriptional responses of the bacterium *Burkholderia terrae* BS001 to the fungal host *Lyophyllum* sp. strain Karsten under soil-mimicking conditions

Irshad Ul Haq* Francisco Dini-Andreote and Jan Dirk van Elsas
Microbial Ecology, Groningen Institute of Evolutionary Life Sciences (GELIFES), Nijenborgh 7, 9747 AG, University of Groningen, The Netherlands.

*Corresponding author: Irshad Ul Haq, Microbial Ecology, Groningen Institute of Evolutionary Life Sciences (GELIFES), Nijenborgh 7, 9747 AG, University of Groningen, The Netherlands.
E–mail address: i.u.haq@rug.nl

Microbial Ecology

Figure S1_A

Distribution of normalized reads number over COG classes

The distribution (normalized) of numbers of reads of transcripts belonging to different COG classes is shown, for treatments B (*B. terrae* BS001, in grey color) and B+F (*B. terrae* BS001 + *Lyophyllum* sp. strain Karsten, in black color). The X axis represents COG classes and the Y axis shows the number of normalized reads. **(a)** T1= day 3, **(b)** T2= day 5 and **(c)** T3= day 8..

COG classes: C– energy production and conversion; E– amino acid transport and metabolism; G– carbohydrate transport and metabolism; H– coenzyme transport and metabolism; I– lipid transport and metabolism; K– transcription; L– replication, recombination and repair; M– cell wall/membrane/envelope biogenesis; O– posttranslational modification, protein turnover, chaperones; P– inorganic ion transport and metabolism; Q– secondary metabolites biosynthesis, transport and catabolism; R– general function prediction; S– functions unknown; T– signal transduction mechanisms; U– intracellular trafficking, secretion, and vesicular transport; V– defense mechanisms.

Figure S1_A

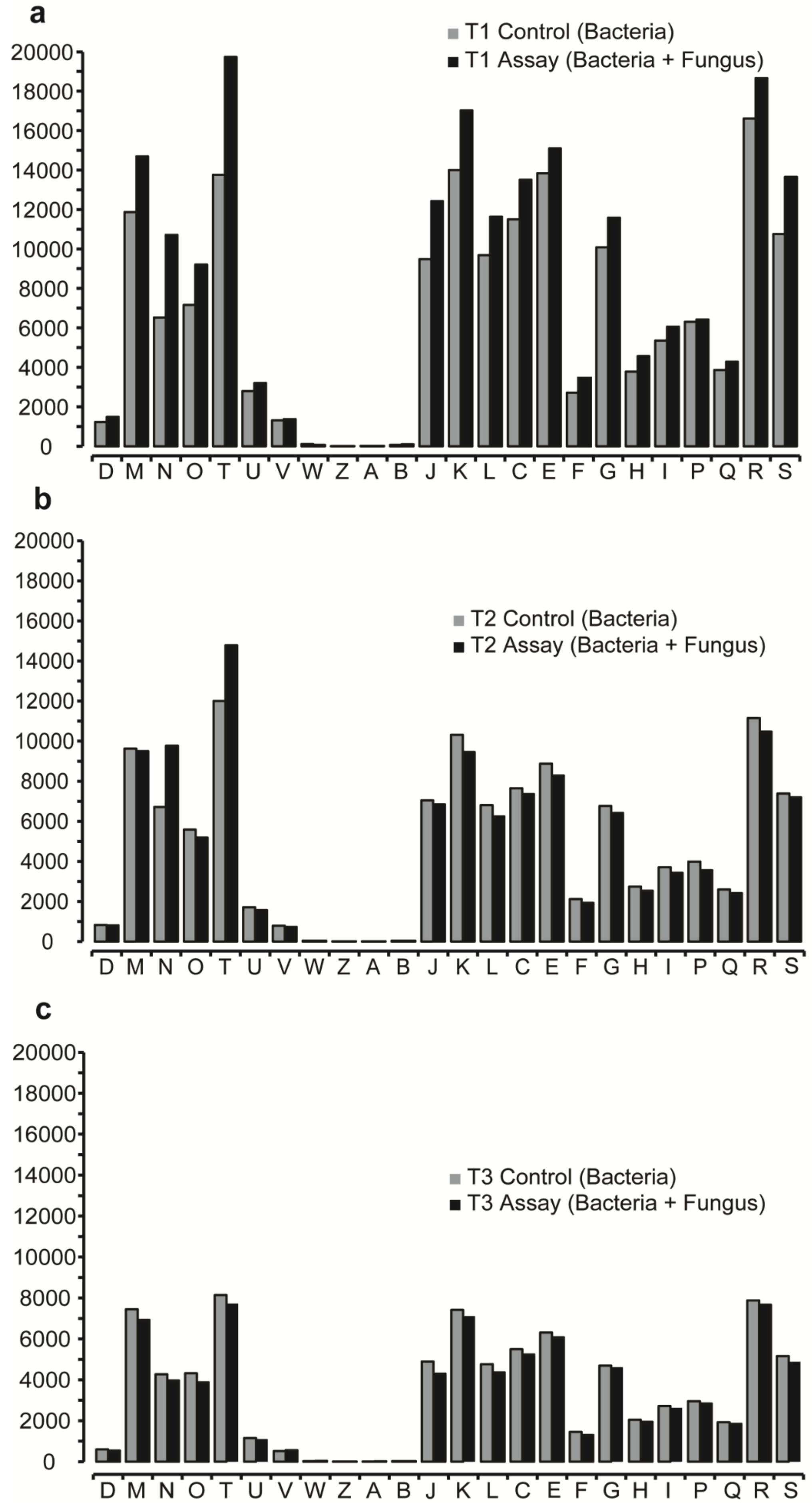
a
T1 Control (Bacteria)
T1 Assay (Bacteria + Fungus)
20000
18000
16000
14000
12000
10000
8000
6000
4000
2000
0
D M N O T U V W Z A B J K L C E F G H I P Q R S
b
T2 Control (Bacteria)
T2 Assay (Bacteria + Fungus)
20000
18000
16000
14000
12000
10000
8000
6000
4000
2000
0
D M N O T U V W Z A B J K L C E F G H I P Q R S
c
T3 Control (Bacteria)
T3 Assay (Bacteria + Fungus)
20000
18000
16000
14000
12000
10000
8000
6000
4000
2000
0
D M N O T U V W Z A B J K L C E F G H I P Q R S

Figure S1_B

Percentage distribution of normalized reads number over COG classes

The percentage distribution of normalized reads of transcripts belonging to different COG classes is shown, for treatments B (*B. terrae* BS001, in grey color) and B+F (*B. terrae* BS001 + *Lyophyllum* sp. strain Karsten, in black color). The X axis represents COG classes and the Y axis shows the number of normalized reads. **(a)** T1= day 3, **(b)** T2= day 5 and **(c)** T3= day 8. The percentage was calculated as the percentage of normalized reads belonging to the indicated COG classes with respect to normalized reads in both treatments.

COG classes: C– energy production and conversion; E– amino acid transport and metabolism; G– carbohydrate transport and metabolism; H– coenzyme transport and metabolism; I– lipid transport and metabolism; K– transcription; L– replication, recombination and repair; M– cell wall/membrane/envelope biogenesis; O– posttranslational modification, protein turnover, chaperones; P– inorganic ion transport and metabolism; Q– secondary metabolites biosynthesis, transport and catabolism; R– general function prediction; S– functions unknown; T– signal transduction mechanisms; U– intracellular trafficking, secretion, and vesicular transport; V– defense mechanisms.

Figure S1_B

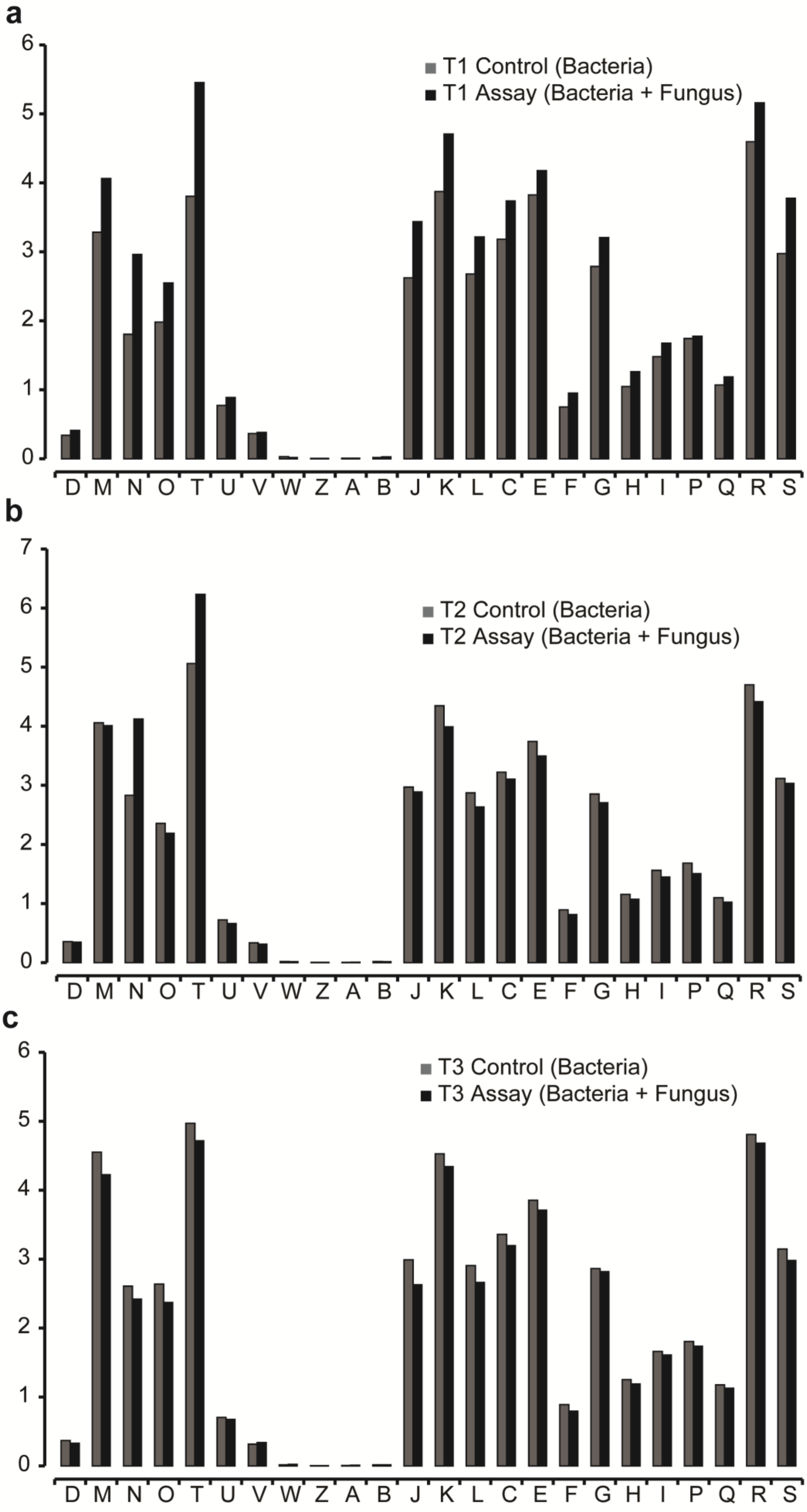
a
T1 Control (Bacteria)
T1 Assay (Bacteria + Fungus)
0 1 2 3 4 5 6
D M N O T U V W Z A B J K L C E F G H I P Q R S
b
T2 Control (Bacteria)
T2 Assay (Bacteria + Fungus)
0 1 2 3 4 5 6 7
D M N O T U V W Z A B J K L C E F G H I P Q R S
c
T3 Control (Bacteria)
T3 Assay (Bacteria + Fungus)
0 1 2 3 4 5 6
D M N O T U V W Z A B J K L C E F G H I P Q R S
