# Supplementary material for: Transcriptional Responses of the Bacterium Burkholderia terrae BS001 to the Fungal Host Lyophyllum sp. Strain Karsten under Soil-Mimicking Conditions
Source: Microb Ecol. 2016 Nov 14;73(1):236–52. doi: 10.1007/s00248-016-0885-7 (PMC5209427; doi:10.1007/s00248-016-0885-7)

Transcriptional responses of the bacterium *Burkholderia terrae* BS001 to the fungal host *Lyophyllum* sp. strain Karsten under soil–mimicking conditions

Irshad Ul Haq* Francisco Dini-Andreote and Jan Dirk van Elsas
Microbial Ecology Group, Groningen Institute of Evolutionary Life Sciences (GELIFES), Nijenborgh 7, 9747 AG, University of Groningen, The Netherlands.

*Corresponding author: Irshad Ul Haq, Microbial Ecology, Groningen Institute of Evolutionary Life Sciences (GELIFES), Nijenborgh 7, 9747 AG, University of Groningen, The Netherlands.
E–mail address: i.u.haq@rug.nl

Microbial Ecology

Figure S2

Volcano plots showing the log2 fold changes (X – axis) with −log10 (*P value*) on the Y – axis for time point 1 (day 3) **(a)**, time point 2 (day5) **(b)** and time point 3 (day8) **(c)**. Dashed vertical lines shows log2 fold change thresholds (>1.5 or <1.5; red dots). Horizontal dashed line shows the boundary line of *P*<0.05.

MA plots showing overall distribution of differentially expressed genes of *B. terrae* BS001 (red dots *P*<0.05) at **d)** time point 1 (day 3), **e)** time point 2 (day5), and **f)** time point 3 (day8), in the presence of *Lyophyllum* sp. strain Karsten. Y – axis represent the log2 fold change (strain BS001 alone and strain BS001 with *Lyophyllum* sp. strain Karsten), whereas the X – axis shows mean of normalized reads counts for both treatments.

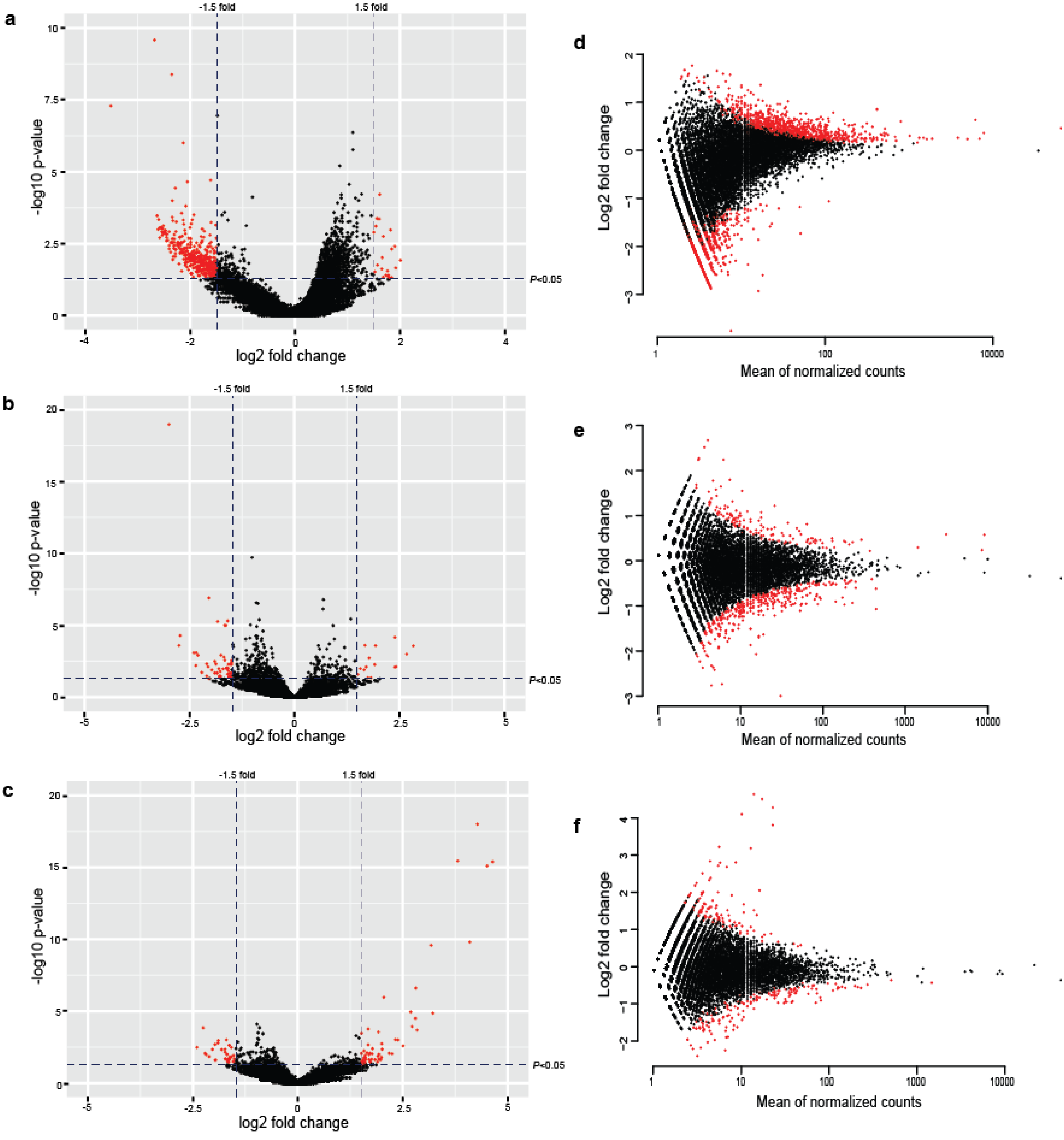

Supplement: Supplementary file 2 — (PDF 228 kb) [file 248_2016_885_MOESM2_ESM.pdf]
