# Supplementary material for: Transcriptional Responses of the Bacterium Burkholderia terrae BS001 to the Fungal Host Lyophyllum sp. Strain Karsten under Soil-Mimicking Conditions
Source: Microb Ecol. 2016 Nov 14;73(1):236–52. doi: 10.1007/s00248-016-0885-7 (PMC5209427; doi:10.1007/s00248-016-0885-7)

Transcriptional responses of the bacterium *Burkholderia terrae* BS001 to the fungal host *Lyophyllum* sp. strain Karsten under soil-mimicking conditions

Irshad Ul Haq* Francisco Dini-Andreote and Jan Dirk van Elsas
Microbial Ecology Group, Groningen Institute of Evolutionary Life Sciences (GELIFES), Nijenborgh 7, 9747 AG, University of Groningen, The Netherlands.

*Corresponding author: Irshad Ul Haq, Microbial Ecology, Groningen Institute of Evolutionary Life Sciences (GELIFES), Nijenborgh 7, 9747 AG, University of Groningen, The Netherlands.
E–mail address: i.u.haq@rug.nl

Microbial Ecology

Figure S3 Three gene clusters potentially involved in the metabolism of oxalate. Heat map showing the expression pattern of genes belonging to different clusters presumed to be involved in the degradation and uptake of oxalate at three different time points (T1, T2 and T3) The heat map was constructed based on normalized read counts. The standardized normalized read counts, denoted as the row Z–score, is plotted in color scale (red indicates higher expression and blue indicates lower expression). The names of the putative gene products are given in front of each gene with their respective locus tags.

Gene clusters potentially involved in oxalate metabolism

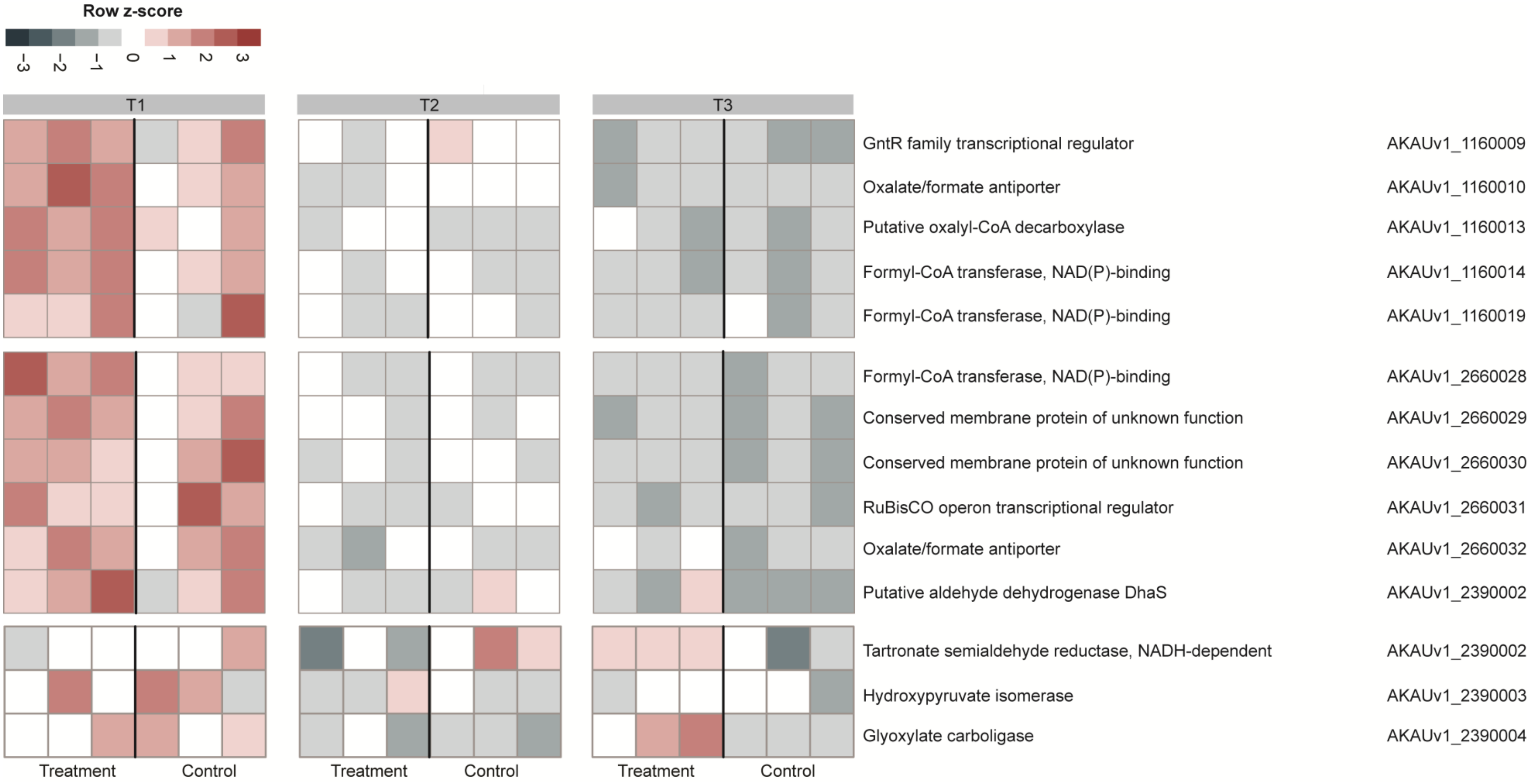

Supplement: Supplementary file 3 — (PDF 266 kb) [file 248_2016_885_MOESM3_ESM.pdf]
