# Supplementary material for: Transcriptional Responses of the Bacterium Burkholderia terrae BS001 to the Fungal Host Lyophyllum sp. Strain Karsten under Soil-Mimicking Conditions
Source: Microb Ecol. 2016 Nov 14;73(1):236–52. doi: 10.1007/s00248-016-0885-7 (PMC5209427; doi:10.1007/s00248-016-0885-7)
Supplement: Supplementary file 4 — (PDF 377 kb) [file 248_2016_885_MOESM4_ESM.pdf]

Transcriptional responses of the bacterium *Burkholderia terrae* BS001 to the fungal host *Lyophyllum* sp. strain Karsten under soil-mimicking conditions

Irshad Ul Haq* Francisco Dini-Andreote and Jan Dirk van Elsas
Microbial Ecology Group, Groningen Institute of Evolutionary Life Sciences (GELIFES), Nijenborgh 7, 9747 AG, University of Groningen, The Netherlands.

*Corresponding author: Irshad Ul Haq, Microbial Ecology, Groningen Institute of Evolutionary Life Sciences (GELIFES), Nijenborgh 7, 9747 AG, University of Groningen, The Netherlands.
E–mail address: i.u.haq@rug.nl

Microbial Ecology

Figure S4 Differentially expressed genes of strain BS001 at T2 (day 5) following confrontation with *Lyophyllum* sp. strain Karsten. a) shows volcano plot, **b)** shows MA plot and **c)** shows bar charts indicating fold changes on log scale for individual genes (red: upregulated, blue: downregulated) grouped into broad functional COG classes, showing differential expression after fungal confrontation (P <0.05; DESeq).

COG classes: C– energy production and conversion; E– amino acid transport and metabolism; G– carbohydrate transport and metabolism; H– coenzyme transport and metabolism; I– lipid transport and metabolism; K– transcription; L– replication, recombination and repair; M– cell wall/membrane/envelope biogenesis; O– posttranslational modification, protein turnover, chaperones; P– inorganic ion transport and metabolism; Q– secondary metabolites biosynthesis, transport and catabolism; R– general function prediction; S– functions unknown; T– signal transduction mechanisms; U– intracellular trafficking, secretion, and vesicular transport; V– defense mechanisms.

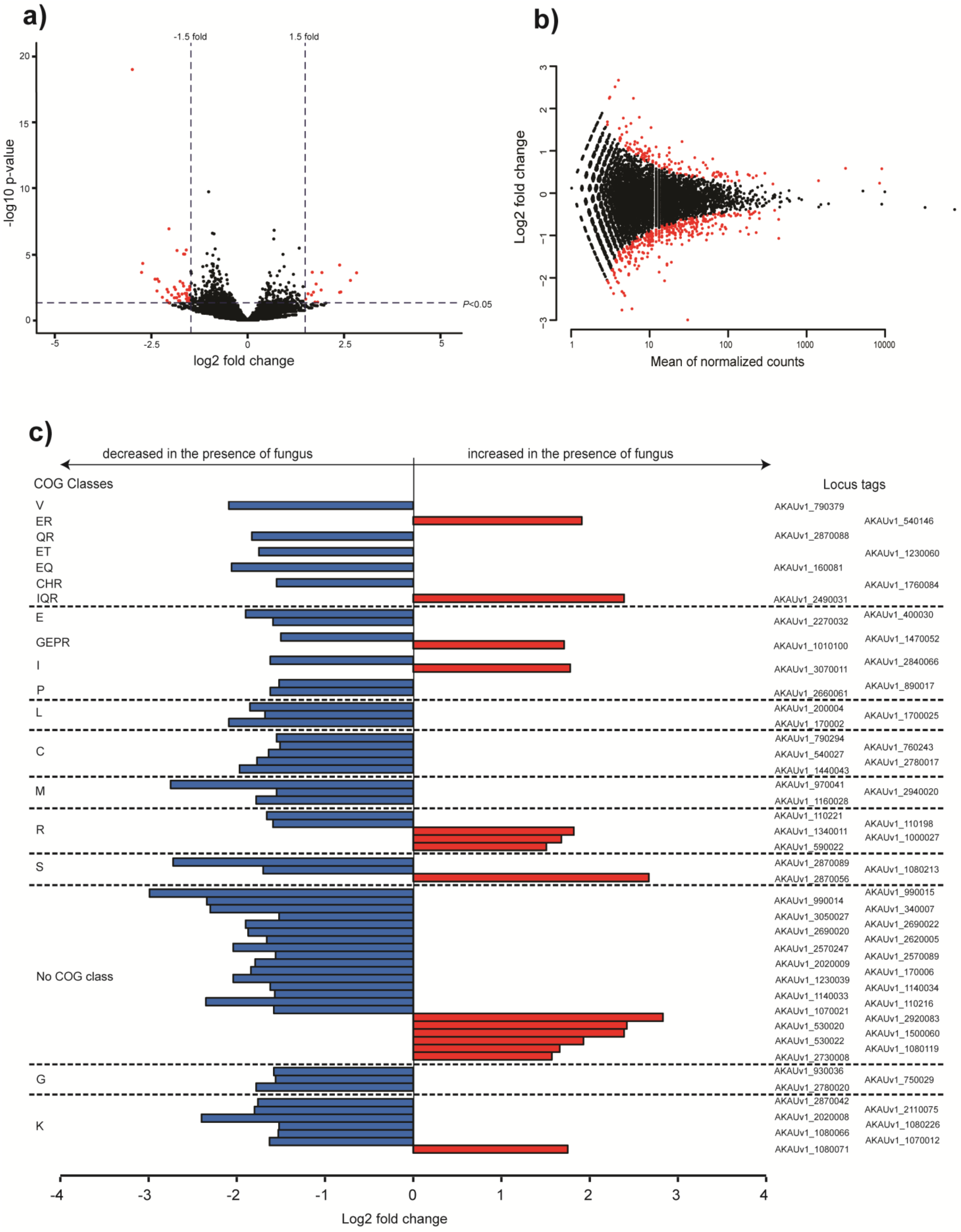
a)
-1.5 fold
1.5 fold
-log10 p-value
20
15
10
5
0
P<0.05
-5
-2.5
0
2.5
5
log2 fold change
b)
Log2 fold change
3
2
1
0
-1
-2
-3
1
10
100
1000
10000
Mean of normalized counts
c)
decreased in the presence of fungus
increased in the presence of fungus
COG Classes
Locus tags
V
ER
QR
ET
EQ
CHR
IQR
E
GEPR
I
P
L
C
M
R
S
No COG class
G
K
AKAUv1_790379
AKAUv1_540146
AKAUv1_2870088
AKAUv1_1230060
AKAUv1_160081
AKAUv1_1760084
AKAUv1_2490031
AKAUv1_400030
AKAUv1_2270032
AKAUv1_1470052
AKAUv1_1010100
AKAUv1_2840066
AKAUv1_3070011
AKAUv1_890017
AKAUv1_2660061
AKAUv1_200004
AKAUv1_1700025
AKAUv1_170002
AKAUv1_790294
AKAUv1_760243
AKAUv1_540027
AKAUv1_2780017
AKAUv1_1440043
AKAUv1_970041
AKAUv1_2940020
AKAUv1_1160028
AKAUv1_110221
AKAUv1_110198
AKAUv1_1340011
AKAUv1_1000027
AKAUv1_590022
AKAUv1_2870089
AKAUv1_1080213
AKAUv1_2870056
AKAUv1_990015
AKAUv1_990014
AKAUv1_340007
AKAUv1_3050027
AKAUv1_2690022
AKAUv1_2690020
AKAUv1_2620005
AKAUv1_2570247
AKAUv1_2570089
AKAUv1_2020009
AKAUv1_170006
AKAUv1_1230039
AKAUv1_1140034
AKAUv1_1140033
AKAUv1_110216
AKAUv1_1070021
AKAUv1_2920083
AKAUv1_530020
AKAUv1_1500060
AKAUv1_530022
AKAUv1_1080119
AKAUv1_2730008
AKAUv1_930036
AKAUv1_750029
AKAUv1_2780020
AKAUv1_2870042
AKAUv1_2110075
AKAUv1_2020008
AKAUv1_1080226
AKAUv1_1080066
AKAUv1_1070012
AKAUv1_1080071
-4
-3
-2
-1
0
1
2
3
4
Log2 fold change
