# Supplementary material for: Transcriptional Responses of the Bacterium Burkholderia terrae BS001 to the Fungal Host Lyophyllum sp. Strain Karsten under Soil-Mimicking Conditions
Source: Microb Ecol. 2016 Nov 14;73(1):236–52. doi: 10.1007/s00248-016-0885-7 (PMC5209427; doi:10.1007/s00248-016-0885-7)
Supplement: Supplementary file 5 — (PDF 360 kb) [file 248_2016_885_MOESM5_ESM.pdf]

Transcriptional responses of the bacterium *Burkholderia terrae* BS001 to the fungal host *Lyophyllum* sp. strain Karsten under soil-mimicking conditions

Irshad Ul Haq* Francisco Dini-Andreote and Jan Dirk van Elsas
Microbial Ecology Group, Groningen Institute of Evolutionary Life Sciences (GELIFES), Nijenborgh 7, 9747 AG, University of Groningen, The Netherlands.

*Corresponding author: Irshad Ul Haq, Microbial Ecology, Groningen Institute of Evolutionary Life Sciences (GELIFES), Nijenborgh 7, 9747 AG, University of Groningen, The Netherlands.
E–mail address: i.u.haq@rug.nl

Microbial Ecology

Fig. S5 Differentially expressed genes of strain BS001 at T3 (day 8) following *Lyophyllum* sp. strain Karsten confrontation. a) shows volcano plot, **b)** shows MA plot and **c)** shows bar charts that are indicating fold changes on log scale for individual genes (red: upregulated, blue: downregulated) grouped into broad functional COG classes, showing differential expression after fungal confrontation (*P* <0.05; DESeq).

COG classes: C– energy production and conversion; E– amino acid transport and metabolism; G– carbohydrate transport and metabolism; H– coenzyme transport and metabolism; I– lipid transport and metabolism; J– Translation, ribosomal structure and biogenesis; K– transcription; L– replication, recombination and repair; M– cell wall/membrane/envelope biogenesis; O– posttranslational modification, protein turnover, chaperones; P– inorganic ion transport and metabolism; Q– secondary metabolites biosynthesis, transport and catabolism; R– general function prediction; S– functions unknown; T– signal transduction mechanisms; U– intracellular trafficking, secretion, and vesicular transport; V– defense mechanisms.

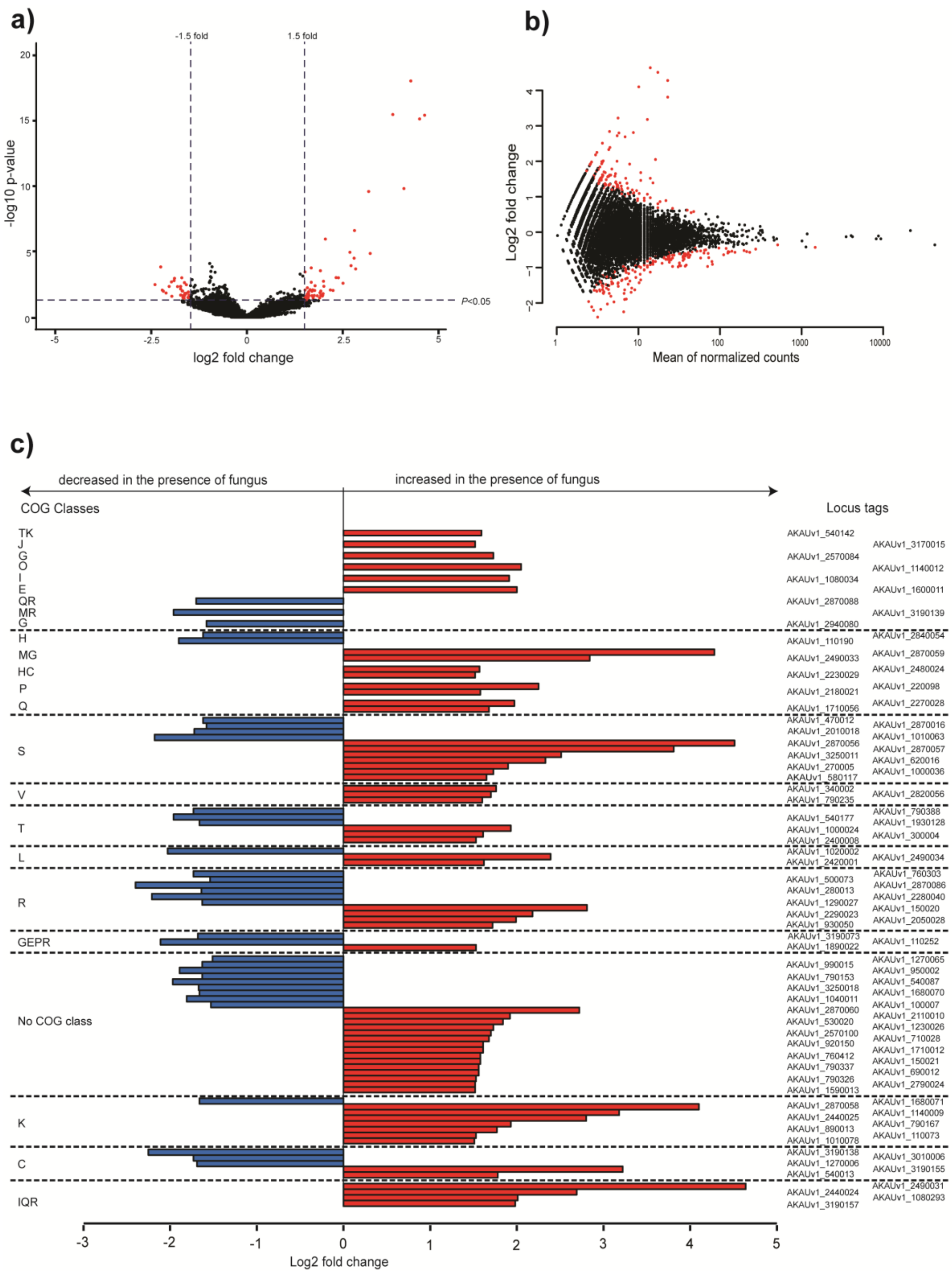

a)
-1.5 fold
1.5 fold
-log10 p-value
20
15
10
5
0
P<0.05
-5
-2.5
0
2.5
5
log2 fold change
b)
Log2 fold change
4
3
2
1
0
-1
-2
1
10
100
1000
10000
Mean of normalized counts
c)
decreased in the presence of fungus
increased in the presence of fungus
COG Classes
Locus tags
TK
J
G
O
I
E
QR
MR
G
H
MG
HC
P
Q
S
V
T
L
R
GEPR
No COG class
K
C
IQR
AKAUv1_540142
AKAUv1_3170015
AKAUv1_2570084
AKAUv1_1140012
AKAUv1_1080034
AKAUv1_1600011
AKAUv1_2870088
AKAUv1_3190139
AKAUv1_2940080
AKAUv1_2840054
AKAUv1_110190
AKAUv1_2870059
AKAUv1_2490033
AKAUv1_2480024
AKAUv1_2230029
AKAUv1_220098
AKAUv1_2180021
AKAUv1_2270028
AKAUv1_1710056
AKAUv1_470012
AKAUv1_2870016
AKAUv1_2010018
AKAUv1_1010063
AKAUv1_2870056
AKAUv1_2870057
AKAUv1_3250011
AKAUv1_620016
AKAUv1_270005
AKAUv1_1000036
AKAUv1_580117
AKAUv1_340002
AKAUv1_2820056
AKAUv1_790235
AKAUv1_790388
AKAUv1_540177
AKAUv1_1930128
AKAUv1_1000024
AKAUv1_300004
AKAUv1_2400008
AKAUv1_1020002
AKAUv1_2490034
AKAUv1_2420001
AKAUv1_760303
AKAUv1_500073
AKAUv1_2870086
AKAUv1_280013
AKAUv1_2280040
AKAUv1_1290027
AKAUv1_150020
AKAUv1_2290023
AKAUv1_2050028
AKAUv1_930050
AKAUv1_3190073
AKAUv1_110252
AKAUv1_1890022
AKAUv1_1270065
AKAUv1_990015
AKAUv1_950002
AKAUv1_790153
AKAUv1_540087
AKAUv1_3250018
AKAUv1_1680070
AKAUv1_1040011
AKAUv1_100007
AKAUv1_2870060
AKAUv1_2110010
AKAUv1_530020
AKAUv1_1230026
AKAUv1_2570100
AKAUv1_710028
AKAUv1_920150
AKAUv1_1710012
AKAUv1_760412
AKAUv1_150021
AKAUv1_790337
AKAUv1_690012
AKAUv1_790326
AKAUv1_2790024
AKAUv1_1590013
AKAUv1_1680071
AKAUv1_2870058
AKAUv1_1140009
AKAUv1_2440025
AKAUv1_790167
AKAUv1_890013
AKAUv1_110073
AKAUv1_1010078
AKAUv1_3190138
AKAUv1_3010006
AKAUv1_1270006
AKAUv1_3190155
AKAUv1_540013
AKAUv1_2490031
AKAUv1_2440024
AKAUv1_1080293
AKAUv1_3190157
-3
-2
-1
0
1
2
3
4
5
Log2 fold change
